# *“Coronavirus Changed the Rules on Everything”*: Parent Perspectives on How the COVID-19 Pandemic Influenced Family Routines, Relationships and Technology Use in Families with Infants

**DOI:** 10.3390/ijerph182312865

**Published:** 2021-12-06

**Authors:** Rebecca Hood, Juliana Zabatiero, Desiree Silva, Stephen R. Zubrick, Leon Straker

**Affiliations:** 1 School of Allied Health, Curtin University, Perth 6102, Australia; juliana.zabatiero@curtin.edu.au (J.Z.); L.straker@curtin.edu.au (L.S.); 2 Telethon Kids Institute, Perth 6009, Australia; desiree.silva@telethonkids.org.au (D.S.); stephen.zubrick@telethonkids.org.au (S.R.Z.); 3 School of Medicine, The University of Western Australia, Perth 6009, Australia; 4 School of Medicine & Health Sciences, Edith Cowan University, Perth 6027, Australia

**Keywords:** children, COVID-19, family relationships, mobile touch screen device use, screen time, technology use, thematic analysis, qualitative research, the ORIGINS Project

## Abstract

This study explores how the first wave of the COVID-19 pandemic influenced family routines, relationships and technology use (smartphones and tablet computers) among families with infants. Infancy is known to be an important period for attachment security and future child development, and a time of being susceptible to changes within and outside of the family unit. A qualitative design using convenience sampling was employed. A total of 30 mothers in Perth, Western Australia participated in semi-structured interviews by audio or video call. All mothers were parents of infants aged 9 to 15 months old. Interviews were audio-recorded and transcribed, and data were analysed using thematic analysis to code and identify themes in an inductive manner. Families described staying home and stopping all external activities. Three themes relating to family interactions and wellbeing were found: enhanced family relationships; prompted reflection on family schedules; and increased parental stress. Two themes related to family device use were found: enabled connections to be maintained; and source of disrupted interactions within the family unit. Overall, participants described more advantages than downsides of device use during COVID-19. Findings will be of value in providing useful information for families, health professionals and government advisors for use during future pandemic-related restrictions.

## 1. Introduction

The novel coronavirus disease (COVID-19) was declared a global pandemic on March 12, 2020 by the World Health Organisation [1]. Globally, countries have experienced varying degrees of infections and deaths that have occurred across multiple ‘waves’. A variety of responses have been adopted to these waves aimed at minimising public health and economic impacts, including social distancing, travel limitations and periods of restrictions (‘lockdown’) which typically require people to stay at home with exceptions for purchasing essential supplies, attending medical appointments, exercise and essential work.

Around the world, the pandemic and its associated restrictions have been linked with social isolation, psychological distress and post-traumatic stress symptoms [2,3,4,5,6]. For example, a US national study of parents with children aged under 18 years found that almost a third of parents reported a decline in mental health for themselves, and 14% reported a decline in behavioural health for their children since the pandemic began [7]. A negative impact of the pandemic on physical activity [8,9], sleep quality [10] and eating behaviours [11] has also been described. For example, a longitudinal study of Croatian adolescents found a significant decrease in physical activity levels during COVID-19 pandemic related restrictions compared to pre-pandemic levels [12].

Although the literature has focused primarily on effects of the pandemic on individual health, theories of family systems suggest that how the COVID-19 pandemic affects one individual may impact the functioning of all family members [13] including parent-child, sibling and marital relationships, as well as connections with extended family and friends. Key frameworks relevant for exploring influences on the family system include attachment theory [14,15], family systems theory [16], the bioecological model [17] and the human-computer interaction model [18,19].

**Figure 1:**
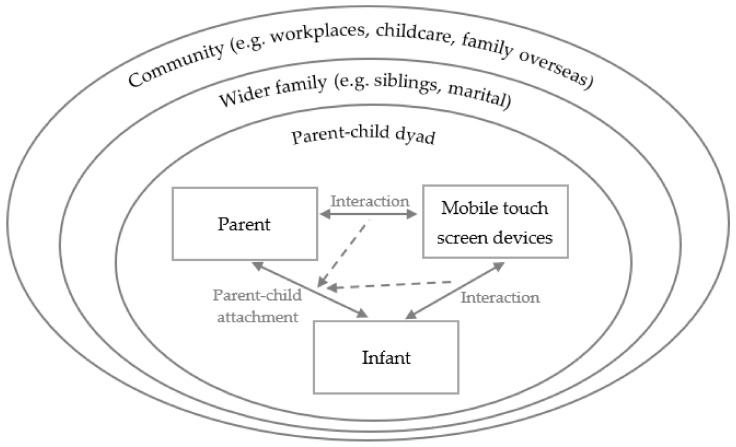
Mobile touch screen device use in an integrated family system.

Figure 1 shows an integrated family systems model of mobile touch screen device use, with solid line arrows depicting the interaction and flow of information. The double headed arrow between parent or infant and mobile touch screen device represents the parent/infant sending information to the device (e.g., opening an App) and the device sending information to the parent/infant (e.g., the device playing music or a guided meditation). The dashed line arrows represent the potential influence of parent-device interaction or infant-device interaction on parent-child attachment.

The integration of these theories acknowledges the likely intertwining of experiences and actions in the parent-child context (e.g., poorer parental mental health due to isolation, fear of infection or financial insecurity), wider family contexts (e.g., disruptions to work and childcare routines) and community contexts (e.g., family living overseas or witnessing the unfolding of the pandemic globally on news outlets). An important assumption is that links within the integrated model are not one-directional, but rather changes to any domain may result in effects to other parts of the model. The disruptions caused by COVID-19 present a unique opportunity to explore the flow-on effects of disturbing typical family routines on how the different parts of the integrated model relate to each other.

The emerging research on the influence of the COVID-19 pandemic on family systems has generally demonstrated more adverse than beneficial effects. For example, a cross-sectional Singaporean study of 258 parents of children aged 12 years and under reported a negative association between parental perceived impact of COVID-19 and parent-child closeness, which was mediated by parental stress [20]. Participants were surveyed during the ‘circuit-breaker’ lockdown period during the first wave of the pandemic in Singapore, when schools and workplaces were closed. In a large cross-sectional study of 4891 Chinese adults in Hong Kong, perceived harms to family well-being due to the COVID-19 outbreak, including increased family negative emotion and decreased family happiness, outweighed the perceived benefits of improved family hygiene, physical health and ability to cope with difficulties [21]. The study was conducted after the second wave in China was under control. A qualitative analysis exploring experiences among families with children aged below four years during the initial COVID-19 lockdown in both Australia and the UK found both positive impacts (including relaxed routines, quality time with family, and positive impact on child development) and negative impacts (including financial insecurity, mental health and not seeing friends and family) [22]. It is important to note that COVID-19 case numbers and government responses (e.g., mandatory mask wearing and enforced lockdown periods) have varied across countries and regions which may influence the impact on families.

It is imperative to extend research in this field given the continued duration of the pandemic with subsequent waves occurring worldwide, and due to the potential for short-term changes in the family system as a consequence of the pandemic leading to longer-term changes. Infancy is known to be a sensitive period for the shaping of future brain function and behaviour based on early experiences [23]. In particular, it is critical to investigate implications of the pandemic on the parent-child relationship among families with infants, given the known importance of developing attachment security in the first years of life for future attachment security [24] and future child development [25,26,27]. Initial research into the role of the parent-child relationship during the COVID-19 pandemic has found attachment security to be a protective factor for adolescent depression and anxiety symptoms during the March 2020 outbreak of COVID-19 in China [28], which is an early indication of the importance of secure parent-child relationships in pandemic conditions.

One of many potential key factors that may be influenced by the COVID-19 restrictions and may affect parent-child relationships and the family system is technology use (in particular mobile touch screen devices such as smartphones and tablet computers) by parents and children. Prior research demonstrates that how parents and children interact with technology is important in whether it enhances child development, such as improving literacy skills [29], or leads to reduced academic achievement [30]; and whether it enhances family connectedness [31] or leads to poorer quality parent-child interactions [32]. A recent systematic review found a very limited number of studies exploring associations between time spent using devices by parents and/or children and parent-child attachment [33] highlighting a need for more quality evidence in this area, including from qualitative research, to better understand potential impacts of device use on parent-child attachment. It is important to explore the potential for COVID-19 to influence both family device use and attachment relationships in order to better understand ramifications of the pandemic and inform appropriate responses.

For many parents and children, the use of devices is a regular part of their lives. For example, three-year-old Canadian children have been found to spend an average of 1.5 h a day using screens (including TV viewing, gaming and mobile devices) [34], and one-third (36%) of Australian pre-schoolers are reported to own their own tablet or smartphone [35]. Many parents often use their devices while supervising children, as evidenced by an observational study which found the majority (76%) used their mobile device while caring for young children in a playground [36]. During the first wave of the global pandemic, both parent and adolescent technology use has been reported to increase for the purposes of communication and distance learning [37,38]. However, little research has explored the influence of COVID-19 on the use of technology by very young children. Given the high level of technology engagement by parents and young children and the importance of parent-child relationships in non-pandemic situations, exploring how technology use and relationships within families of young children have been influenced by the COVID-19 pandemic will provide valuable information.

Prior research indicates a potential bi-directional relationship, whereby higher parent-child interaction scores have been found to predict less future child device use, as well as less child time spent using device use predicting more nurturing future parenting [39]. This bi-directional relationship is likely to apply to pandemic conditions due to increased time together and increased technology use during periods of restrictions.

This study aimed to extend research into how the COVID-19 pandemic has resulted in changes to the family system, specifically family routines, relationships and technology use by parents and infants. The findings will be of value in providing useful information for families, health, education and social welfare professionals and government advisors for use during future pandemic-related restrictions.

## 2. Materials and Methods

### 2.1. Study Design

A qualitative design involving semi-structured interviews with a convenience sample of Western Australian parents was used. Participants were from the larger longitudinal birth cohort study titled the ORIGINS Project (https://originsproject.telethonkids.org.au, accessed on 6 December 2021) which invited participation by families who were 18 weeks pregnant, when attending private and public health services at a tertiary hospital in Perth, Western Australia.

This study is a sub-project of the ORIGINS Project. This unique long-term study, a collaboration between Telethon Kids Institute and Joondalup Health Campus, is one of the most comprehensive studies of pregnant women and their families in Australia to date, recruiting 10,000 families over a decade from the Joondalup and Wanneroo communities of Western Australia.

### 2.2. Recruitment

Potential participants for the current study were provided with information about a study on mobile touch screen device use and attachment and with the opportunity to opt-out from any further contact. Those who did not opt-out were recruited via email and phone call if they had an infant aged 9 to 15 months at the time of the interview, had sufficient English proficiency, and were available for a qualitative interview either by audio call or video call (due to COVID-19 restrictions). The target age of the infants of interviewed families was 12 months. However, to enable flexibility with booking and scheduling interviews with participants, the age bracket was extended to three months either side of this age.

Participants were remunerated with an AUD 50 voucher for participation in the study. Ethics approval was provided by Joondalup Health Campus Human Research Ethics Committee (approval # 1804) and Curtin University Human Research Ethics Committee (approval # AHRE2018-0065).

### 2.3. COVID-19 Context

**Figure 2:**
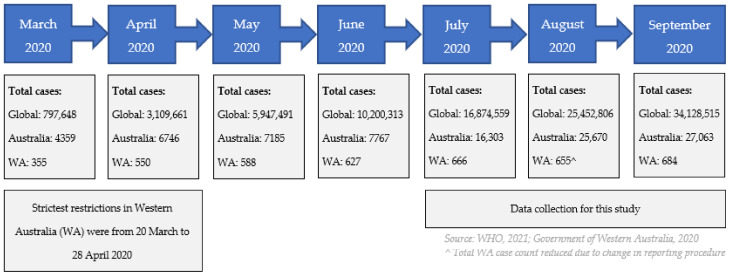
Total COVID-19 case numbers at the end of each month, key time-points and study design.

When recruitment commenced for this study at the end of July 2020, there were close to 17 million total COVID-19 cases and over 700,000 deaths globally, and around 17,000 total cases and 200 deaths in Australia [40] (see Figure 2). In Western Australia, the setting for the current study, there were low case numbers and deaths (665 cases and 9 deaths).

The strictest restrictions for Western Australia were in place between 20 March and 28 April 2020. This included closure of Australian borders to non-residents, closure of schools, non-essential venues and activities, and prohibition of regional travel [41,42]. Residents were required to stay at home with exemptions for purchasing essential supplies, attending medical appointments, one-hour daily exercise, commuting to work if unable to work from home and compassionate reasons [43].

It is important to note that this study was conducted at a time of uncertainty (with vaccines in early stages of development) and prior to subsequent waves of the pandemic for many countries, including subsequent waves in the Eastern States of Australia. Western Australia was well placed to deal with the repercussions of the pandemic due to a well-equipped and equitably accessible healthcare system, a strong economy supported by resource production, capacity for strong border control due to Australia being an island and government financial supplements provided to business and employees affected by the pandemic. Despite low case numbers at the time of the study, there was a great deal of uncertainty about the progression of the pandemic and whether case numbers would escalate rapidly, as well as if an effective vaccine would become available in the future. Therefore, strict restrictions were enforced.

### 2.4. Data Collection/Instrument

An interpretive description framework [44] was used to generate data with practical outcomes. A semi-structured interview schedule was used, with questions based on findings from previous research on young children’s screen technology use (Supplementary File S1). The schedule was developed in consultation with experts in the field and reviewed by the ORIGINS Project community reference group.

A semi-structured interview format was chosen to enable open-ended questions and the ability to prompt for further information. Reflective listening also enabled an in-depth understanding of participants’ perspectives. Interviews were audio-recorded and transcribed verbatim.

The interview schedule included open-ended questions relating to how COVID-19 influenced family:

(1). routines including work and childcare;
(2). interactions and relationships;
(3). device use by parents and infants.

Interview questions used to evaluate parent-to-infant attachment were adapted from the Maternal Postnatal Attachment Scale [45]. These questions covered the same constructs of postnatal attachment as the quantitative scale, but in a qualitative approach using open-ended questions relating to the parent’s thoughts, feelings and behaviours towards their infant.

### 2.5. Data Analysis

Interview transcriptions were entered into NVivo (QSR International Pty Ltd., Perth, Australia2020) to facilitate organisation and analysis of data. Data were analysed by RH using thematic analysis to code and identify emerging themes in an inductive manner [46]. A second researcher (JZ) independently reviewed RH’s interpretation of the data, to improve trustworthiness of data analysis. Data are reported in accordance with the Consolidated Criteria for Reporting Qualitative Research (COREQ) Checklist [47].

Once interviews were completed, participants were contacted by phone for member checking purposes. Contact was made with 14 participants, who were presented with key themes and asked if they perceived it to be a reasonable summary. All agreed that it was a reasonable summary and provided no new information.

## 3. Results

### 3.1. Sample and Data Collection

At the end of July 2020, there were 282 ORIGINS Project participants who were mothers of infants aged 9 to 15 months and were therefore eligible for this sub-project. A total of 100 potential participants who did not opt-out to further contact received an email invitation and phone call. Of these, 30 were willing and able to participate in the interview, providing a response rate of 30%.

For all 30 interviews, the interview was conducted with the mother. Although interviews were available to either/both parent(s), no fathers participated in the interviews. Interviews were conducted by RH between July 2020 and September 2020, with 16 conducted by phone and 14 by video-call, according to the preference of the interviewee. On average, the length of the interviews was 56 min, ranging from 30 to 76 min.

The mean (range) age of mothers was 34 years (21 to 42 years). The mean (range) age of infants was 12.5 months (9 to 15 months). Most participants (*n* = 28) were married, and all were currently living with the father of the infant. Half of the participants had one child only, and the other half had between two and five children. The older children were aged 3 to 9 years.

Of the 30 participants, 16 were currently working in a full-time, part-time or casual position, and three of these were also studying concurrently. Six participants were employed but on maternity leave. Of the eight participants not currently employed, two had recently been made redundant as a result of the COVID-19 pandemic and were seeking employment. Most husbands/partners (*n* = 28) were employed in a full-time position. One was employed in a part-time role and one in a casual role. Five husbands/partners had Fly-In-Fly-Out work positions where they were rostered to work away from the home for periods of time.

### 3.2. Influence of COVID-19 on Family Routines

The pandemic had a varied effect on family work routines (Table 1). While several participants described no changes to their family’s working arrangements, most stated that they experienced an increase or decrease in working hours, and many described either themselves or their partner working from home for a few weeks to a few months.

For several families, there were no changes to childcare arrangements. However, others described taking their child out of childcare either due to the parent(s) staying home or due to wanting to avoid potential exposure to COVID-19. Most described staying home and stopping all activities. For some, this continued once restrictions eased.

### 3.3. Influence of COVID-19 on Family Relationships

Analysis of the data yielded three key themes in relation to the influence of COVID-19 on family relationships. These themes, displayed in Table 2, were: enhanced family relationships; prompted a reflection on family schedules; and increased parental stress.

Almost a third of mothers described an enhanced relationship with their infant as a result of COVID-19 restrictions due to spending more time at home together. A couple of participants mentioned noticing their child displaying signs of separation anxiety due to the increased time at home. For example, one participant stated: *“I don’t think I noticed as much until after the restrictions started to ease. But his separation anxiety, even the first time we had someone over to the house after all the home isolation type stuff, he completely freaked out. So I think he was clinging to me more than I’d actually realized during that time.” (P17, 35 yo, 15 mo, 3 yo).*

No mothers described poorer attachment with their infants due to COVID-19 restrictions. However, one mentioned her child was less dependent on her since having to swap roles with her husband and increase her hours of work while he stayed at home caring for the children: *“She just wasn’t so dependent on me anymore…It’s been nice for him [husband] and most of the time I think it’s nice, but there’s still part of me that goes, well, she’s my little baby. I liked being her favourite. But you’ve got to do what you’ve got to do.” (P28, 38 yo, 13 mo, 3 yo]:*

Several participants reflected on the benefits that COVID-19 restrictions had on their typical schedules or routines and described enjoying spending additional time together. Some participants described changes to their family interactions including more time engaging in home-based family activities such as playing board games, arts and crafts and riding bikes.

In terms of parental mental health, several participants spoke of the lockdown situation as being overwhelming and stressful due to uncertainty about the future, having the children home for an extended period of time with no break, and not being able to leave the house. However, one participant noted less worry for her and her husband, explaining that: *“We actually found it great…because of the postnatal depression he [husband] was happier because he wasn’t as worried about me and how I was coping and things like that, because he could be here. COVID made that time easier for us.” (P30, 35 yo, 12 mo, 3 yo).*

Many of the mothers interviewed talked of feeling isolated and alone due to decreased opportunities to connect socially with other parents, and less face-to-face interactions and support from family members either residing locally or interstate/overseas. The repercussions of the restrictions on sustaining early friendships were also described.

### 3.4. Role of Mobile Touch Screen Device Use during COVID-19

#### 3.4.1. Time Using Devices

Around a third of participants described no changes to their family device use during the COVID-19 pandemic. Almost two-thirds described an increase in the use of devices, typically in relation to their own use of them. For example, one mother described: *“I’ve gotten a lot worse since COVID. Before, I didn’t really use to spend a lot of time on my phone, but now I’m finding that I am on it way more than usual.” (P20, 32 yo, 11 mo, 3 yo).* Another participant stated: *“Coronavirus changed the rules on everything. So I probably wouldn’t have expected us to be using the devices as much, but then no one expected a pandemic either…The rule book went out the window.” (P15, 42yo, 14mo, no other children).*

A couple of participants described less time using devices. For example: “*While we were all at home, we kind of made more of an effort to spend more time with the family. So we did a lot of board games and colouring in and activities and stuff rather than, we didn’t have much time on the tablets or TV or anything at all.” (P2, 29 yo, 10 mo, 6 yo)*.

#### 3.4.2. Reasons for Using Devices

Most important reasons for using devices more during COVID-19 restrictions were:

- Communication with family and friends, especially family interstate and overseas;
- Keeping children entertained while at home;
- Home-schooling and educational apps;
- Exercise such as yoga classes on YouTube;
- Online shopping;
- Working from home such as meetings via Zoom;
- Appointments such as physiotherapy;
- Reading news about the pandemic; and
- Searching for ideas for home activities to do with children.

Boredom was also a common reason for using devices more frequently or for longer periods. For example, one mother described: “*Because we’re stuck in the house a bit more, I guess I get more bored and just pick up the phone and start looking through it rather than being out doing other stuff.”* (*P4, 38yo, 11mo, no other children*). Another stated: *“I was never an online shopper before [COVID] and I didn’t actually need most of the things I bought, but you just, a lot of was out of boredom or just having not gone out.”* (*P28, 38 yo, 13 mo, 3 yo*).

Several participants described continuing with increased device use once restrictions had eased. For example: *“I probably am still using it a little bit more because it became a bit more of a habit. So yeah, there’s times where I pick up my phone to check it and I realize that I’ve only just really put it down. I don’t know. It’s probably become a bit of a mindless kind of thing. It’s out of habit. I’ll finish what I’m doing and pick up my phone and it may have only been a couple of minutes.” (P15, 42yo, 14mo, no other children)*. Another participant reflected: *“That’s the downside. I developed this habit of being on my phone and it hasn’t really gone away.”* (P20*, 32 yo, 11 mo, 3 yo).*

#### 3.4.3. Influence of Device Use

A couple of participants stated that the increased use of devices during COVID-19 had no effect on other aspects of their lives. For participants who did describe an impact, analysis of the data yielded two key themes in relation to the influence of COVID-19 on family device use: maintained connections, and disrupted interactions within the family unit (Table 3).

Around half of the participants spoke of the importance of devices in maintaining communication with family and friends around Australia and overseas. This was particularly important for families with relatives residing in countries with higher rates of COVID-19 cases and deaths. In contrast to the benefits participants described of device use during COVID-19, one participant stated that she felt *“very frustrated and overwhelmed with all the messaging from friends…it stressed me out.” (P29, 35 yo, 12 mo, 3 yo).*

The ability to continue with activities was described as a beneficial aspect of device use, with participants using devices to maintain a sense of normalcy and continue with activities during the lockdown period such as baby sensory classes, martial arts, exercise classes and church mass. A couple of participants described this ability to connect with the outside world and continue activities as stress relieving.

A few participants mentioned their devices being a source of distraction within the family unit during the pandemic, due to spending more time on them during the period of restrictions.

## 4. Discussion and Implications

In-depth interviews with 30 mothers of infants aged 9 to 15 months found that COVID-19 restrictions had substantial and varied effects on family routines, relationships and technology use. The proposed model of family human-computer interaction in a COVID-19 context that was based on concepts of attachment theory [14,15], family systems theory [16], the bioecological model [17] and human-computer interaction [18,19] provides a useful framework for investigating perceived influences and reveals a potential cascading effect. The ‘circuit breaker’ lockdown approach, which has been found to be an advantageous response for reducing public health and economic impacts in relation to COVID-19 outbreaks for high income countries such as Australia [48], had flow-on effects to the community, wider family and parent-child levels of the integrated model. The role of technology use varied at each level.

At a community level, a varied effect of COVID-19 on parents’ workplace and childcare arrangements was described, demonstrating that family systems were uniquely and heterogeneously influenced by the pandemic at this level. Interviewed parents reported disruptions to forming early connections with other local mothers, which led to feelings of isolation and loneliness. However, feelings of isolation were mitigated to a degree by the ability to maintain connection with extended family and friends and continue activities via the use of mobile touch screen devices. This highlights the positive role of device use during COVID-19 at the community level in alleviating feelings of isolation and loneliness. These findings indicate that devices have played a vital role within the proposed model by enabling continued communication and engagement in activities in a virtual capacity during pandemic-related restrictions. This is similar to findings of an Italian cross-sectional study, where keeping in touch with family and friends via a virtual setting was perceived to be a useful strategy for maintaining social relationships and mitigating the psychological effects of lockdown [49]. 

At the wider family and parent-child levels, positive and negative effects on relationships were described. This supports findings of a qualitative study of family functioning during the COVID-19 lockdown period in Spain, which revealed themes related to both improvement (including family (re)connection, better communication and emotional expressiveness) as well as deterioration themes (including loneliness, family distance and conflict atmosphere) [50]. In addition, a cross-sectional study of 4342 Chinese school students found that around half (52%) perceived the experience of home quarantine as positive for reasons including increased time at home and with parents, and negative impacts including not being able to meet friends and classmates, disturbance in hobbies and interests and disturbance to their regular routine [51]. In the current study, many parents described an enhanced relationship with their infant as a result of the COVID-19 pandemic, which is in line with an Australian survey of 2018 parents of children 18 years and under where almost half (42%) stated they were more connected to their child since the pandemic [52].

In terms of technology use, most families described increased time spent using devices which continued once restrictions eased. This is similar to a study of 2426 Chinese children and adolescents, which found a significant increase of approximately 30 h a week of screen time during the pandemic compared to before the pandemic [53]. Additionally, in a cross-sectional study of 1836 American mothers of pre-schoolers, most (74%) reported an increased in child screen-time due to the COVID-19 outbreak, with screen-time higher in homes that had greater scores of ‘household chaos’ [54]. In an Australian online poll, 51% of families reported an increase in child screen time for entertainment due to the pandemic associated restrictions [50]. Although most parents surveyed (76%) felt screen time had an overall positive effect on their child during this time, ‘excessive screen time’ was reported as the top-rated child health problem [50].

The findings of the current study provide further evidence that family relationships and technology use have been affected by the pandemic, and that using devices in a way that reinforces benefits and reduces downsides is important, especially in a pandemic context.

At the individual level, some mothers interviewed in this study described increased stress due to the COVID-19 pandemic and government-imposed restrictions, including concerns about the future and being overwhelmed with the length of time spent with their children at home without practical support or a break. This is consistent with other studies that have explored psychological effects of lockdown, such as a cross-sectional study of Cypriot adults which found higher perceived stress and lower social support during lockdown [55]. In a U.S. longitudinal study, parental stress (most commonly related to changes in children’s routines and worry about COVID-19) increased from before COVID-19 to during the peak of stay-at-home mandates, and remained elevated once restrictions eased [56]. These findings demonstrate the substantial effect the pandemic had, and is continuing to have, on many parents and families.

These findings suggest that the COVID-19 pandemic influenced the family system in a myriad of ways, and highlights the complexities of disruptions to everyday family life. The influence of device use on family interactions showed differential effects, where benefits and disadvantages were related to the nature of screen use rather than simply the amount of screen use. The use of device for the specific purposes of communication and continuation of activities appeared to enhance connectedness within families, whereas increased general device use led to more opportunities for disrupted interactions within the family unit.

It is important to note that the Western Australian wave-one lockdown was of a relatively short duration, and longer periods of pandemic-related restrictions have been associated with poorer mental health [57] as well as poorer economic growth [58]. Although the same government restrictions applied to all families, the ways with which these restrictions impacted on everyday life and family routines differed, which supports prior suggestions that people have heterogeneous responses to pandemic situations due to reasons such as pre-existing psychopathology [59].

It is also critical to consider long-term implications of the pandemic on family interactions and technology use beyond the easing of restrictions. Detrimental health consequences of COVID-19 lockdown have been found to persist after the lifting of restrictions in longitudinal studies in England [60] and Austria [61]. Therefore, there is the potential for short-term changes in family relationships and technology use due to the COVID-19 pandemic to lead to longer-term changes, and policy should be directed at promoting positive family interactions and screen use behaviours beyond the easing of restrictions.

Although the focus of most research relating to COVID-19 has been on implications to the individual, these findings demonstrate that government-imposed restrictions and lockdown periods are influential to parent-child, wider family and community contexts. Overall, the findings indicate that access to devices has played a positive role in alleviating the effects of the COVID-19 pandemic on families. However, being aware of the potential downsides of technology use in creating new habits and disrupting family interactions is likely to be of value for families in making wise technology use decisions during pandemic related restrictions. Useful information (e.g., recommendations or guidelines) for families should be prepared for use during future pandemic-related restrictions.

## 5. Strengths and Limitations

### 5.1. Strengths

This study adds information to the COVID-19 literature by emphasizing implications for family routines and highlighting complexities surrounding the benefits and downsides related to family relationships and device use. The qualitative interview approach provided rich and detailed information of family experiences during the first wave of the pandemic. The study was well timed in terms of capturing parent reflections within weeks of lockdown restrictions occurring, which may have led to reduced memory bias and also enabled participants to have had time to reflect on their experiences prior to being interviewed.

A further strength of this study was in proposing a model of family human-computer interaction in a COVID-19 context that recognises the importance of considering multiple layers of influences on relationships and device use, and acknowledging that these influences do not occur in isolation but as part of a system.

### 5.2. Limitations

A limitation of this study was that a convenience sample was used which did not include families with a full range of characteristics that could influence device use and attachment e.g., single parents, infants with insecure attachments, families with poor marital relationships. This study was conducted in Perth, Western Australia where the lockdown period was relatively short and case numbers/deaths were relatively low. In addition, Western Australia fared better than most other advanced national economies. In addition, government approaches differ interstate as well as internationally which may limit generalizability of the findings.

It is important to consider that there are potentially other influences that may not have been captured by this cohort of Western Australian mothers, and their reflections may not apply to the experiences of all people in all situations across all times. For example, the findings suggest families without digital access may have fared worse during the pandemic given the positive effects of technology use in alleviating feelings of isolation and enabling activities to continue; however, this could not be studied in this cohort as all families interviewed had access to mobile devices and the internet. In addition, the study captured a snapshot in time, and does not explore long-term effects of the pandemic and related restrictions.

## 6. Future Research

Given the dynamic nature of the COVID-19 pandemic, continued research into the impacts on family routines, relationships and technology use is essential in appropriately addressing the needs of children and parents throughout ongoing waves of the pandemic.

Future research could explore the impact of device use on child and parent outcomes within different family structures and age groups (toddlers, preschoolers and grade-schoolers) to better inform appropriate technology use decisions during pandemic-related restrictions. Future research could also explore other potential influences of COVID-19 on families such as mental health, family income and education. Extending research in this field would be of benefit in better understanding the implications of the pandemic, and for informing the trialing of interventions to support parents in mitigating the effects of the pandemic on their families, enhancing any potential benefits and being cognisant of potential detriments of device use.

Further research could investigate associations between mobile touch screen device use, parent-infant attachment and child development in general (unrelated to COVID-19) in order to ensure guidelines for the use of devices within families are as appropriate and comprehensive as possible.

## 7. Conclusions

In summary, the findings indicated that:

1. Families described staying at home and stopping all external activities during the strictest pandemic-related restrictions in Western Australia.
2. Three themes relating to family interactions and wellbeing were found due to the pandemic and associated restrictions: enhanced family relationships; prompted reflection on family schedules; and increased parental stress.
3. Two themes related to family device use were found: enabled connections to be maintained; and source of disrupted interactions within the family unit.
4. Overall, participants described more advantages than downsides of device use during COVID-19.
5. Findings will be of value in providing useful information for families, health professionals and government advisors for use during future pandemic-related restrictions.

## Figures and Tables

**Table 1:** Influence of COVID-19 on family routines.

Theme	Sub-Theme	Participants	Representative Quotes
Changes to parent(s) work hours	Parent(s) had increased hours	1, 11, 12, 21, 28	◦ P11 [35yo (owns a sales business), 14mo, 4yo]: “When the pandemic hit, I now have to work more because I’ve got less staff hours. I’m working a lot more than I was planning on unfortunately. It’s the only way to do it.” ◦ P28 [38yo (works in retail), 13mo, 3yo]: “Our routine was completely out the window and I was just working extra when needed and I would be kind of on call, a bit of relief work.”
	Parent(s) had reduced hours	9, 16, 18, 19, 23, 29	◦ P29 [35yo, 12mo, 3yo]: “My husband went down to three days. His whole office did to try and save money but not have to let anyone go” ◦ P16 [39yo, 14mo, 4yo]: “For a few months there was a disruption. My husband was working only three days a week.”
	Parent(s) worked from home	3, 12, 14, 18, 21, 22, 25, 27, 30	◦ P3 [31yo, 10mo, no other children]: “My husband did three months working from home” ◦ P21 [35yo, 15mo, no other children]: “Corona threw everything out…We couldn’t go on campus for the first half…I would just hole myself off in the [home] study and pretend I wasn’t there”
	Parent(s) made redundant	5, 13	◦ P5 [26yo (worked in hospitality), 14mo, no other children]: “I’m just a blubbering mess…I got stood down from work…My partner luckily still had an income. Like he was still working. He didn’t get stood down either. So I’m lucky compared to some people. But it doesn’t make it any easier” ◦ P13 [37yo (worked in Human Resources), 11mo, no other children]: “I literally worked up until the week before having bubs and the intention was to go back in September three days a week. It’s what I was hoping, but I was made redundant last month”
Changes to childcare	No changes	2, 7, 8, 10, 26	◦ P10 [39yo, 14mo, no other children]: “I felt it was a safe thing to do” ◦ P26 [33yo, 12mo, 3yo, 5yo]: “It was family daycare so we just kept going through Corona. We weren’t interrupted at all which was good”
	Commenced daycare	1	◦ P1 [21yo, 9mo, no other children]: “The boys went into daycare for the first time while it was free” (note: Child care services were fee-free for families between 6 April and 28 June 2020 in Western Australia)
	Abstained from daycare	9, 11, 16, 20, 30	◦ P9 [32yo, 13mo, no other children]: “When it was quite bad in Western Australia around March/April, we just had to take him out of daycare for that two months and we would just stay at home” ◦ P16 [39yo, 14mo, 4yo]: “We didn’t send our son to daycare because my parents came over from abroad and they are 70 years old, so we didn’t want to take a risk.”
Changes to other activities	Stopped usual activities and stayed home during lockdown	1, 3, 4, 13, 15, 24, 28	◦ P1 [21yo, 9mo, no other children]: “We basically didn’t leave the house for two months” ◦ P4 [38yo, 11mo, no other children]: “We didn’t go to the library or rhyme time or catch up as much with other mums.” ◦ P13 [37yo, 11mo, no other children]: “Because of the isolation restrictions it meant that we weren’t able to go and do the activities that we were doing such as baby sensory and Gymbaroo…which meant being stuck at home which became very much a bit of a ground hog day”
	Continued to reduce activities after lockdown restrictions eased	3, 15, 28	◦ P3 [31yo, 10mo, no other children]: “We haven’t re-joined any of our classes that we did before. So it’s still a bit, we’re still a bit wary when we go out.” ◦ P15 [42yo, 14mo, no other children]: “Even before they started asking people to stay at home, I decided that’s one thing that we could do for the community is to stay home as much as possible. So we probably, we did stay home more and we are still staying home more.” ◦ P28 [38yo, 13mo, 3yo]: Before COVID happened we had a busier week where we had swimming lessons and we would go to a dance class. But once that all stopped we just haven’t got back into it.”

**Table 2:** Influence of COVID-19 on family interactions and wellbeing.

Theme	Sub-Theme	Participants	Representative Quotes
Enhanced family relationships	Enhancing relationship between mother and infant	4, 9, 16, 17, 18, 22, 23, 26	◦ P4 [38yo, 11mo, no other children]: “We’ve become more attached because we’ve had to stay at home together.” ◦ P17 [35yo, 15mo, 3yo]: “It did affect our relationship. In a good way. We were quite close.” ◦ P18 [36yo, 12mo, no other children]: “It actually made me more connected to him because I worked from home so I had time and he could see me throughout the day.”
	Enhancing relationship between father and infant	19, 28	◦ P19 [32yo, 11mo, no other children]: “Since this COVID-19 started my husband is at home more, so that’s when I see that he’s getting more closer to his dad.” ◦ P28 [38yo, 13mo, 3yo]: “She [1 year old] was with him [father] twenty-four seven. So they did become more attached, which was quite nice.”
	Enhanced relationship within family unit	2, 13, 16, 22, 23, 30	◦ P23 [29yo, 12mo, 3yo]: “I’d probably say it brought us all [mother, father and two children] closer to be honest because we had to entertain them as opposed to going out and entertaining them, like at the aquarium or the zoo. Like I had to entertain them at home. So yeah, I guess it brings that bond closer.” ◦ P13 [37yo, 11mo, no other children]: “With my husband, he had to work from home for about six weeks. And because of that, it allowed us to go for a walk together in the morning and we were able to have lunch together. And that was actually really really nice. And that was definitely nice for the [marital] relationship.” ◦ P30 [35yo, 12mo, 3yo]: “It actually was a blessing in lots of ways as well. It was good family time and actually being able to properly interact with each other at home…There was more free time to actually do things and activities. So probably in that way, it was probably better for our [family] relationship.” ◦ P16 [39yo, 14mo, 4yo]: “I think the Corona, talking to other people and feeling it for myself, it actually deepens the family bond because you realize that, either spending more time at home or just listening to other stories, it feels like: ‘This is important. I don’t need to go anywhere really.’”
Prompted a reflection on family schedules	Reflection on family schedules	6, 16, 22, 30	◦ P6 [38yo, 13mo, no other children]: “It was nearly a welcome change because it gave us an excuse to stay at home…You kind of had more time to yourself that you could concentrate on their development rather than rushing around trying to do these classes. And now I kind of realize after the fact that we probably do a little bit too much. It was probably nice to actually get to take the break, and less is more with babies. I think I’ve just learned that.” ◦ P16 [39yo, 14mo, 4yo]: Before I used to go shopping because I didn’t know what to do with myself and I needed to get out of the house, and now I don’t have that need anymore and I feel like this is good, we can just be us…I think it brought us closer. The value of spending time together and that’s the time we’ve got, we should be spending together and enjoying it…It actually teaches you things, teaches you to embrace your family.” ◦ P22 [41yo, 12mo, 4yo]: “Coronavirus kind of reaffirmed the need for healthy habits and finding a nice balance. You know, finding, trying to find a balance between the benefits of using screen time, using screens to promote how you live rather than letting screen times dictate how you live.”
	Changes to family interactions	2, 13, 21, 22, 23	◦ P22 [41yo, 12mo, 4yo]: “We did a lot more…We were throwing balls, riding bikes, playing board games, my little one would help me with sewing.” ◦ P21 [35yo, 15mo, no other children]: “I bought some art supplies and things like that so we could do more activities together.” ◦ P23 [29yo, 12mo, 3yo]: “We would go for walks every day, and yeah, generally we try and do different activities.”
Increased parental stress	Parental stress	1, 5, 13, 20, 29	◦ P29 [35yo, 12mo, 3yo]: “I did feel a bit crazy maybe…Starts to get full on looking after the two kids, without them being at daycare and getting that break. It gets overwhelming and stressful.” ◦ P5 [26yo, 14mo, no other children]: “For me to not be able to get out of the house and do something it really *** with me. Sorry for my language. My mental health side of it went down because it’s not like I could just go out and do what I wanted.” ◦ P1 [21yo, 9mo, no other children]: “It did become very stressful because we were worried. Obviously through all the media with everything going on which was all coming through the devices that we were going to, he was going to lose his job and it was going to be the end of the world. So it became very stressful for all of us, finding articles and sending it to him, you know, and him, us wanting to protect our son, make sure that we will have food, you know?”
	Social isolation	13, 15, 18, 20, 21, 24, 27	◦ P27 [41yo, 13mo, no other children]: “I felt very alone and very isolated... It triggers all those feelings of isolation and just endless hours at home by myself or with [child].” P13 [37yo, 11mo, no other children]: “The mother’s group had only met a couple of times. And so to then just not meet, like our mother’s group essentially is kind of diminished because there wasn’t long enough, strong enough connections to keep it going. And I feel sorry for the people who didn’t get to join the mother’s group at all, because I desperately needed that support at the beginning.” ◦ P24 [34yo, 12mo, 3yo, 5yo]: “I felt a bit sorry for him (1 year old) because it was going to be the time that he started doing his little groups and things at the beginning of COVID. And then they were all cancelled so he didn’t.” ◦ P20 [32yo, 11mo, 3yo]: “It was so hard because I see my parents quite a lot, and they refused to see me. My husband was at work and it was pretty much just me and the kids all day, every day.” ◦ P21 [35yo, 15mo, no other children]: “My family’s all on the East coast [of Australia] so they haven’t been able to come over and visit for our birthday. And my mum is, this is her first grandchild, so she’s really missing seeing everything. She would have been over here at least once, maybe twice if it wasn’t Corona.”

**Table 3:** Role of mobile touch screen device use during COVID-19.

Theme	Sub-Theme	Participants	Representative Quotes
Maintained connections	Enabling communication with family	3, 7, 10, 13, 15, 16, 17, 18, 21, 22, 25, 26, 27	◦ P10 [39yo, 14mo, no other children]: “It was very bad in Italy and it was a bit overwhelming for our family back there. So we tried to call them a little bit more because they had the lockdown. So they had to stay home and we tried to be more close to them and call them more often. So maybe we called even more than once a day.” ◦ P15 [42yo, 14mo, no other children]: “We had a number of family birthdays over Zoom.” ◦ P16 [39yo, 14mo, 4yo]: “Using devices actually helped us to get in touch. We probably got in touch more often than we normally do, just to check on them [family in Italy] and [ask] “is everything all right and how are you coping being under lockdown?” I think with the rest of the family, actually devices and all the Skype messages and the emails helped, and sending photos of grandchildren to keep the spirits up and things like that. That was actually a good thing. I think it came handy and we used it in a positive way.” ◦ P22 [41yo, 12mo, 4yo]: “We do more video calls now because we can’t go and visit family in the Eastern States (of Australia).” ◦ P27 [41yo, 13mo, no other children]: “All our family is in England which aren’t dealing with the pandemic very well. So it’s been really nice to check in with them.”
	Enabling activities to continue	7, 13, 15, 17, 21, 22, 24, 25	◦ P13 [37yo, 11mo, no other children]: “So in particular with Corona, because we had been doing baby sensory classes we’re doing some online videos through the restrictions. So I did a little bit of that with him, which helped with the bonding at the time.” ◦ P15 [42yo, 14mo, no other children]: “Even our physio exercise classes were on Zoom. We had our church mass on Zoom… They [devices] probably helped both of our mental states…For me to be able to do some exercise and everything and just see that there was other people out there, that life was going on.” ◦ P21 [35yo, 15mo, no other children]: “It helped alleviate a bit of our anxiety, just living through a pandemic…By having something to still be connected to the rest of the world I think it was stress relieving.” P25 [31yo, 12mo, 8yo]: “Martial arts went online. So we actually did that from home for a while there, which was pretty cool.” ◦ P7 [39yo, 13mo, 4yo, 9yo, 9yo, 9yo]: “They [9 year old daughters and their friends] do bingo online with each other and that (you know, even just talking online), that was a whole new thing. And that’s a positive thing.”
Disrupted interactions within family unit	Increasing distraction from family	4, 11, 20, 23	◦ P4 [38yo, 11mo, no other children]: “Instead of going out to catch up with friends or stuff like that, we have to do it on the phone, which means increased phone use and more distractions from each other.” ◦ P20 [32yo, 11mo, 3yo]: “I was maybe spending more time on my phone than with him, I suppose. If he was happy exploring a room, then I would just be there, but then I’d be on my phone just keeping an eye on him. I suppose I wasn’t really interacting with him.” ◦ P23 [29yo, 12mo, 3yo]: “With the pandemic we probably gave them [children] like more screen time. So then they wouldn’t be interacting I guess with each other.” ◦ P11 [35yo, 14mo, 4yo]: “I guess the only thing is most probably again, the distraction. More time equals more screen time. Like more time at home equals more screen time.”

## Supplementary Materials

The following are available online at https://www.mdpi.com/article/10.3390/ijerph182312865/s1, Supplementary File S1: Interview Schedule.
