# Supplementary material for: “Coronavirus Changed the Rules on Everything”: Parent Perspectives on How the COVID-19 Pandemic Influenced Family Routines, Relationships and Technology Use in Families with Infants
Source: Int J Environ Res Public Health. 2021 Dec 6;18(23):12865. doi: 10.3390/ijerph182312865 (PMC8657679; doi:10.3390/ijerph182312865)
Supplement: Supplementary file 1 [file ijerph-18-12865-s001.zip › ijerph-1455419-supplementary.pdf]

Supporting information - *"Coronavirus changed the rules on everything":* Parent perspectives on how the COVID-19 pandemic influenced family routines, relationships and technology use in families with infant – by Hood et al

File S1. Interview Schedule

Prior to initiating the interview: Researcher introduces themselves, gives a summary of the project aim and procedures (including audio recording), clarifies any queries participant may have about the study, provides definitions for terms used (e.g. screen devices) and obtains participant consent to be interviewed and for the information we collect as part of this study to be shared with the ORIGINS Databank.

1. Can you tell me about your family?
    a. Where do you live?
    b. Who lives with you? (e.g. adults and marital status, children (gender, age))
    c. Working status for yourself and your partner (if applicable), school/kindy status for children (if applicable), typical weekly routines (work/school/kindy) (pre-pandemic)
    d. **Have your family's work/child care arrangements changed as a result of the COVID-19 pandemic?**

2. Can you tell me about the type of screen devices you and your family have in the home?
    a. How many screen devices and what type?
    b. Where these screen devices are located in the home?
    c. Who has access to the screen devices and when?
    d. Are any of these screen devices used outside of the home (e.g. car trips, shops, work/school, parks, family and friends' houses)

3. Can you tell me what a typical week of screen device use would look like for you and each of your family (partner and child(ren) if applicable)?
    a. Let's start with your week – on Mondays what devices do you use in the morning….are the other week days similar? Is your use of screens different on Saturday? on Sunday?
        i. Home vs outside of the home (work/school)?
        ii. What types of programmes or activities/apps are watched/done with each screen device and by whom?
        iii. How are the screen devices used (individually/collaboratively)?
        iv. How do you feel about your family's current screen use practices?
        v. How has your family's technology use practices changed from pregnancy to now? Is your family's current use of technology different to what you expected it to be?
        vi. **Has your family's use of screen devices changed as a result of the COVID-19 pandemic?**

4. Can you tell me about the reasons why you and your family use screen devices?
    a. What do you and your family use the screen devices for?
        i. You, partner, each child (if applicable)
        ii. What do you and your family expect from the use of screen devices?

5. Can you tell me more about how you and your family manage the use of screen devices?
    a. Have you considered or discussed any strategies you and your family use to decide how or when to use screen devices?
        i. If so, can you tell me more about it (who developed them? How are they used?)
    b. What else has influenced your decisions around screen use?

6. We would like to better understand what your relationship is like with your infant.
    a. What can you tell me about your relationship with your child? (e.g. how you think and feel towards your child? How you behave towards your child?)
    b. How has your relationship with your child changed from pregnancy to now?
    c. What do you think helps you connect with your child?
    d. What do you think hinders you from being connected with your child?
    e. **Has your relationship with your child changed as a result of the COVID-19 pandemic?**

7. We would like to know your thoughts on how the use of screen devices, particularly mobile touchscreen devices, by you and/or other members of your family may influence, in any way…
    a. The relationship between you and your child? e.g. how you think and feel towards your child? How you behave towards your child? What screen device use practices help you connect with your child? What screen device use practices distract you from being connected with your child?
    b. The interactions between the family members?
        i. e.g. <u>You and your partner/family members other than children</u>: how you think/feel/behave towards each other; how much time you spend together
        ii. e.g. <u>Your partner and your child(ren)(if applicable)</u>: how he/she thinks/feels/behaves towards the child; how much time he/she spends with the child
        iii. e.g. <u>Your children (if applicable)</u>: how they think/feel/behave towards each other; how much time they spend together
    c. How do you think the influence of device use on relationships in your family has changed from pregnancy to now? Do you think the influence of device use on relationships is different to what you expected it to be?
    d. **How do you think the influence of device use on the relationship between you and your child (and other relationships within your family) has changed as a result of the COVID-19 pandemic?**

8. We would like to know your thoughts on how the use of screen devices, particularly mobile touchscreen devices, by you and/or other members of your family may influence, in any way…
    a. How your child(ren) learns (e.g. how they explore the environment, learn to solve problems, copy/mimic your actions such as scribbling with a pen on paper)
    b. How your child(ren) communicates with other people (e.g. play games such as peekaboo, clap hands, wave bye-bye, says words other than mama and dada, points at objects, hugs a doll or stuffed animal)
    c. How your child(ren) develops physically (e.g. how they learn to hold different objects, throw a ball, turn pages of a book, sit/crawl/stand up/walk)
    d. How do you think the influence of device use on how your child is developing these skills has changed as a result of the coronavirus?
    e. **How do you think the influence of device use on how your child is developing these skills has changed as a result of the COVID-19 pandemic?**

9. What kind of information would you find useful to help guide your family's use of mobile touch screen devices?
    a. How would you like to receive that information? (e.g. online seminar, brochure, through your playgroup)
